# Light Quality Potentiates the Antioxidant Properties of *Brassica rapa* Microgreen Extracts against Oxidative Stress and DNA Damage in Human Cells

**DOI:** 10.3390/antiox12101895

**Published:** 2023-10-23

**Authors:** Ida Paolillo, Giulia Costanzo, Antonella Delicato, Filippo Villano, Carmen Arena, Viola Calabrò

**Affiliations:** 1 Department of Biology, University of Naples Federico II, Via Cinthia, 80126 Napoli, Italy; ida.paolillo@unina.it (I.P.); giulia.costanzo2@unina.it (G.C.); antonella.delicato@unina.it (A.D.); fil.villano@studenti.unina.it (F.V.); vcalabro@unina.it (V.C.); 2 NBFC—National Biodiversity Future Center, 90133 Palermo, Italy

**Keywords:** antioxidants, phytochemicals, human cells, DNA damage, NRF2, p21WAF proteins

## Abstract

Plants are an inexhaustible source of bioactive compounds beneficial for contrasting oxidative stress, leading to many degenerative pathologies. *Brassica rapa* L. subsp. *rapa* is well known for its nutraceutical properties among edible vegetable species. In our work, we aimed to explore an eco-friendly way to enhance the beneficial dietary phytochemicals in this vast world of crop-growing plants at selected light quality conditions. White broad-spectrum (W) and red–blue (RB) light regimes were used for growing brassica microgreens. The organic extracts were tested on keratinocytes upon oxidative stress to explore their capability to act as natural antioxidant cell protectors. Our results show that both W and RB extracts caused a notable reduction in reactive oxygen species (ROS) levels induced by H_2_O_2_. Interestingly, according to its higher contents of polyphenols and flavonoids, the RB was more efficient in reducing ROS amount and DNA damage than the W extract, particularly at the lowest concentration tested. However, at higher concentrations (up to 100 μg/mL), the antioxidant effect reached a plateau, and there was little added benefit. These findings confirm that RB light effectively increases the antioxidant compounds in *Brassica rapa* L. microgreens, thus contributing to their enhanced activity against oxidative-induced genotoxicity compared to microgreens grown under W light.

## 1. Introduction

Consumption of foods rich in bioactive compounds has been associated with positive effects on human health, acting on inflammation and diseases such as cancer and diabetes, as supported by many preclinical and clinical studies [1,2]. Edible green vegetables, including herbal medicines, are well-established sources of phytochemicals [3] which are crucial components in promoting human health and longevity [4]. Bioactive compounds are often found in edible plant extracts and are known for their antioxidant and anti-inflammatory properties. Natural antioxidants can counteract tissue inflammation and oxidative stress, which are underlying factors in developing and progressing systemic diseases such as metabolic disorders, cardiovascular diseases, neurodegenerative diseases, and cancer [5]. Unlike synthetic antioxidants, natural compounds from edible plants play a vital role in protecting cells with minimal or no side effects [6]. Therefore, the demand for plant-based antioxidants in pharmaceuticals and nutraceuticals is continuously increasing. This trend reflects the growing interest in using plants as sources of phytochemicals to promote health and prevent or manage various health conditions.

Brassicaceae plants are vegetables consumed all over the world [7]. *Brassica* is a source of healthful compounds, i.e., polyphenols, vitamins, fiber, soluble sugars, glucosinolates minerals, and carotenoids [8]. More specifically, the health benefits of *Brassica* are related to its antioxidant capacity due to the presence of many phenolic compounds [9], phenolic acids, flavonoids, and isoflavonoids. The main phenolics in *Brassica* are hydroxycinnamic acids, i.e., caffeic, ferulic, protocatechuic and sinapic acids [10]. These health-promoting compounds are involved in numerous biological activities, such as cancer prevention [11], cardiovascular system protection, and inhibition of oxidative damage [12]. Considering the importance of therapeutic consumption of this family of vegetables, it represents an innovative scientific frontier to develop an eco-friendly strategy to increase the concentration of antioxidants, including flavonoids and phenolic compounds, in *Brassica* species destined for human consumption.

Light is one of the critical factors that can be modulated to influence the accumulation of these metabolites. Several studies showed that light quality significantly impacts on plant growth and chemical composition [13,14,15,16,17].

The modulation of the light spectrum through Light-Emitting Diode (LED) technology enables obtaining desired plant characteristics in plants, i.e., growth enhancement, changes in morphology, flavor and pigmentation, and the stimulation of specific bioactive molecule synthesis.

Therefore, monochromatic light or a mix of different wavelengths may be effectively used to affect plant material/extract with customized composition for various purposes such as medicinal, nutritional, and industrial applications [16].

Recent studies proved that red and blue regions of the light spectrum are the most effective in influencing plant growth and phytochemical composition [18,19,20]. The use of red–blue light (RB) and white full-spectrum light (W) has distinct effects on plants. It has been reported that RB and W treatments affected the synthesis of polyphenols, anthocyanins, and flavonoids in some fruits and vegetables [21,22].

More specifically, as described by [19], RB light regimes increased the concentration of some flavones, flavanols, anthocyanidins, and benzoic and cinnamic acids in citrus fruits compared to W regime. The more specific qualitative analysis of the phenolic profile in fruits showed that RB light boosted some phenolic substances such as sinensetin, linarin, caffeic, ferulic, and vanillic acids, known for their anti-inflammatory, antimicrobial, cardioprotective, anticancer, and antidiabetic properties. On the other hand, fruits stored under W light showed an increased concentration of other metabolites, i.e., flavonoids such as rutin, quercetin, and naringin, effective in treating neurodegenerative diseases, COVID-19, and metabolic syndrome, respectively. 

However, even if combining red and blue light can yield impressive results, it is crucial to adjust the ratio of these light regimes appropriately as well as the light intensity [23]. Excessive blue light, indeed, may lead to a decrease in yield, showing the importance of finding the right balance between the two wavelengths [24,25]. The present work aims to investigate the possibility of enhancing the antioxidant compounds of *Brassica rapa* L. microgreens growing plants under RB light regime and test the corroborated extracts for their antioxidant potential on non-transformed human cells. More specifically, we have treated human immortalized keratinocytes with different concentrations of the brassica crude extracts from plants grown at RB and W light regimes to explore their activity against hydrogen peroxide-induced oxidative stress in human cells.

In our experiments, the cruciferous species *Brassica rapa* L. (Broccoli raab) and, more specifically, broccoli microgreens were used for preparing the crude extracts. *Brassica* microgreens are young plants already known as a fount of valuable bioactive compounds [26,27] showing rapid growth and adaptability to environmental factors [28].

## 2. Materials and Methods

### 2.1. Chemicals and Reagents

Folin and Ciocalteu’s phenol reagent, sodium carbonate, aluminium chloride hexahydrate, sodium nitrite, sodium hydroxide, 2,4,6-tri(2-pyridyl)-s-triazine (TPTZ), iron (III) chloride hexahydrate and all standards (gallic acid, catechin, 6-hydroxy-2,5,7,8-tetramethylchroman-2-carboxylic acid, 97% (Trolox)) and Dymethil Sulfoxide (DMSO) were purchased from Sigma-Aldrich (St. Louis, MO, USA). All other chemicals used were of analytical grade. Dulbecco’s Modified Eagle’s Medium (DMEM, Sigma Chemical Co., St. Louis, MO, USA), FBS, Hyclone Laboratories, Inc. Logan, UT, USA, 3-(4,5-methylthiazol-2)-2,5-diphenyltetrazolium bromide (MTT, neoFroxx Einhausen Deutschland, Germany), Dimethyl Sulfoxide for cell culture, Applichem GmbH ITW Reagents, Glenview, IL, USA, 2′-7′ dichlorofluorescein diacetate (DCFDA, Sigma Chemical Co., St. Louis, MO, USA), Hydrogen peroxide solution 30%, Carlo Erba Reagents GmbH, Milan, Italy, Trolox, 6-Hydroxy-2,5,7,8-tetramethylchroman-2-carboxylic Acid (Sigma Chemical Co., St. Louis, MO, USA), PBS Dulbecco’s Phosphate-Buffered Saline (Euroclone S.p.A, Milan, Italy), DAPIsolution (1:50,000) (Sigma Chemical Co., St. Louis, MO, USA), Dako mounting medium (Agilent, Santa Clara, CA, USA), anti-YB-1 (Abcam 12148, Cambridge, UK), anti-g-H2AX histone (Cell signaling 9718S, Danvers, MA, USA), Alexa Fluor 488 anti-rabbit donkey and Alexa Fluor 594 donkey (Thermo Fisher Scientific, Waltham, MA, USA), anti-β-actin (C4) and anti-NRF-2 (A-10) were from Santa Cruz Biotechnology (Dallas, TX, USA), while p21WAF were from Invitrogen (Thermo Fisher Scientific, Waltham, MA, USA).

### 2.2. Experimental Design and Plant Material 

**Figure 1:**
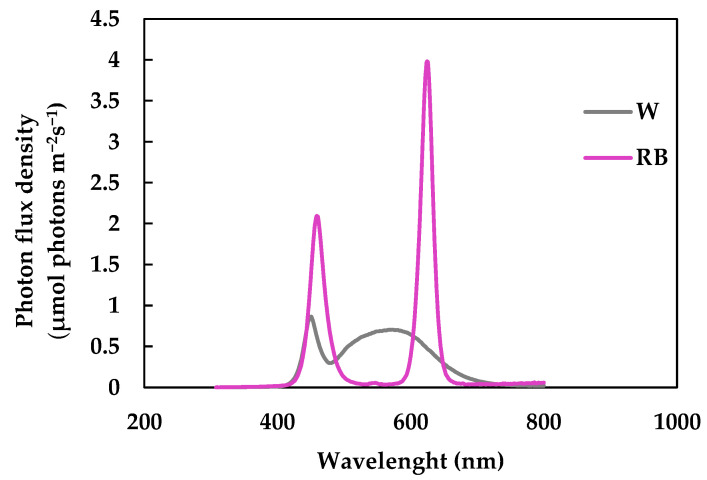
Light quality treatments applied to plants in chamber: white (W) and red-blue (RB), with a red emission peak at 630 nm, and a blue emission peak at 460 nm.

One hundred seeds of *Brassica rapa* L. subsp. *rapa* were sown in two 0.5 L boxes filled with Vigorplant© complete universal soil (21% Baltic peat 0–10 mm, 37% Irish peat 0–20 mm, slow-release fertilizer, 13% volcanic pumice 3–8 mm, 29% superfine peat 0–3 mm). The seeds were placed in a chamber at the following controlled condition: Photosynthetic Photon Flux Densities (PPFD) 270 ± 10 mmol(photon) m^−2^ s^−1^, 25/15° day/night temperature, 50/70% day/night relative humidity, at 12 h photoperiod. The light quality regimes set in the chamber were: broad-spectrum white (W) and red–blue (RB: 60–40) with emission peaks of 630 and 460 nm for red and blue, respectively (Figure 1). 

Temperature and RH% were recorded by a digital thermo-hygrometer (HC520 Digital Thermo-Hygrometer, Cheerman, Shenzhen, China) while PPFD was measured by a quantum sensor (Li-Cor, Lincoln, NB, USA). Microgreens were left in the chamber for fifteen days and regularly watered to reintegrate water lost by evapotranspiration.

### 2.3. Preparation of Methanolic Extracts

Plant material, collected at 15 DAS, was finely powered with liquid nitrogen by mortar and pestle. The extracts were prepared by weighing 0.20 g of sample, solubilized in 2 mL of 80% methanol. After centrifugation at 11.000 rpm samples were stored at 4 °C until analysis.

### 2.4. Polyphenols and Flavonoids Determination

Total polyphenols were evaluated as described by Sun et al. (1998) [29]. An aliquot of methanolic extract was mixed with 1:1 (*v*/*v*) 10% Foli–-Ciocálteu and 1:5 (*v*/*v*) 700 mM Na_2_CO_3_ and the solution was stored in the dark for 2 h. The absorbance was detected by a spectrophotometer (UV-VIS Cary 100, Agilent Technologies, Palo Alto, CA, USA) at 765 nm. The total polyphenol content was calculated by means of a gallic acid standard curve and expressed as mg Gallic Acid Equivalents (GAE) g^−1^ FW. 

Flavonoids were quantified accordig to Moulehi et al. (2017) and Dewanto et al. (2002) [30,31]. Briefly, an aliquot of supernatant was added to 75 μL of 5% NaNO_2_ (sodium nitrite). After, 150 μL of 10% AlCl_3_ (aluminum chloride) and 500 μL NaOH (1 M), distilled water was added to the mixture up to a final volume of 1.525 mL. The absorbance was quantified spectrophotometrically (spectrophotometer UV-VIS Cary 100, Agilent Technologies, Palo Alto, CA, USA) at 510 nm. Flavonoid concentration was estimated using a catechin standard curve as mg Catechin Equivalents per gram of fresh weight (mg CE g^−1^ FW). Statistical analysis was performed on 8 technical replicates by *t*-test, using GraphPad Prism8 software Prism software, version 8.0 (GraphPad, San Diego, CA, USA). 

### 2.5. Antioxidant Activity Assay

The antioxidant capacity was evaluated by FRAP (Ferric Reducing Antioxidant Power) assay, as reported by George et al. (2004) [32]. Briefly, 0.25 g of powdered sample was extracted with 5 mL of methanol/water solution (60:40 *v*/*v*). After 1 h on ice, the extracts were centrifuged at 4 °C for 15 min at 14.000 rpm. After, an aliquot of 150 µL t was added to the FRAP reagents (2.5 mL of 300 mM acetate buffer pH 3.6, 250 µL of 10 mM triperidoltriazine (TPTZ), and 250 µL of 12 mM FeCl_3_), and stored in the dark for 1 h. The absorbance was detected at 593 nm spectrophotometrally (UV-VIS Cary 100, Agilent Technologies, Palo Alto, CA, USA). The antioxidant capacity was calculated using a Trolox standard curve and expressed as µmol Trolox equivalents (µmol TE g^−1^ FW). Statistical analysis was performed on 8 technical replicates by *t*-test, using GraphPad Prism software, version 8.0 (GraphPad, San Diego, CA, USA). 

### 2.6. Cell Culture, Reagents, and Preparation Samples

HaCaT keratinocytes were provided from the Service Cell Line (GmBH, Eppelheim, CLS, Germany). They have spontaneously immortalized from adult skin having mutant p53 (H179Y/R282W) and are still able to differentiate upon calcium treatment. HaCaT cells were cultured in Dulbecco’s Modified Eagle’s Medium (DMEM, Sigma Chemical Co, St. Louis, MO, USA) added with 10% fetal bovine serum (FBS, Hyclone Laboratories, Inc. Logan, UT, USA) in a humified atmosphere of 5% CO_2_. at 37 °C cells were grown at 60–70% for subculturing and routinely evaluated for mycoplasma contamination. After W and RB methanolic extracts have been dried, at a temperature of 50 °C for 30′ in Rotavapor RII (BÜCHI Labortechnik AG ^®^, Flawil, Switzerland), and dry material was weighed. The extracts were solubilized in Dimethyl Sulfoxide (DMSO) was added to reach the concentration of 1000 µg/mL (stock solution). 

From stock solution, serial dilutions were added to the HaCaT cell culture medium to obtain the final concentrations of 100 µg/mL, 10 µg/mL and 1 µg/mL. 

The DMSO concentration in negative control and treated samples was maintained at 0.1% in both 3-(4,5-methylthiazol-2)-2,5-diphenyltetrazolium bromide (MTT) and Dichlorofluorescein diacetate (DCFDA) assays.

### 2.7. Cell Viability (MTT) Assay

The effect of *Brassica rapa* extracts on cell viability was assessed on HaCaT cells measuring the reduction of 3-(4,5-methylthiazol-2)-2,5-diphenyltetrazolium bromide (MTT) to blue/purplish formazan salts by the mitochondrial enzyme succinate dehydrogenase. The experiment was carried out according to [33]. 

We performed the cell count at 24 and 48 h. Every 24 h, cells were rinsed with 1× PBS, trypsinized, and counted by Scepter 2.0 Handheld Automated Cell Counter (Millipore, Burlington, MI, USA) as previously described [34]. Statistical analysis was performed on six technical replicates by one-way ANOVA, followed by Dunnett’s multiple comparisons test using GraphPad Prism software, version 8.0 (GraphPad, San Diego, CA, USA).

### 2.8. Dichlorofluorescein Diacetate (DCFDA) Assay

The antioxidant activity of *Brassica rapa* extracts was measured in HaCaT cells by means of 2′-7′ dichlorofluorescein diacetate (DCFDA), which gives a fluorescent compound following oxidation by Reactive Oxigen Species (ROS). Cells were seeded at 2 × 10^4^ in 96 wells. After 24 h, cells were washed with PBS and pretreated with the extracts at the indicated concentrations for 4 h. The medium was supplemented with 1 mM DCFDA for 45 min, then 1 mM (3%) H_2_O_2_ was added for 1.5 h. The measurement of ROS was obtained using a Sinergy H4 microplate reader (Gen5 2.07). The fluorescence emitted from the cells treated with DCFDA was compared to the cells in 0.1% DMSO as a negative control. Trolox, a Vitamin E analogous, was used as a positive control. 

Statistical analysis was performed on six technical replicates by two-way ANOVA, followed by Tukey’s multiple comparison test using GraphPad Prism software, version 8.0 (GraphPad, San Diego, CA, USA).

### 2.9. Immunofluorescence Microscopy on HaCaT Cells

The 3.0 × 10^4^ HaCaT cells were seeded on coverslips and treated for 24 h. After, both control and treated cell groups were rinsed with × PBS and fixed in PFA 3.7% for 10 and 15′, respectively. Cells were 3-fold washed with PBS and twice with a 3% BSA solution to avoid unspecific binding of antibodies. After 1 h incubation at RT with primary antibodies, anti-YB-1 (Abcam 12148), and anti-γ-H2AX histone (Cell signaling 9718S), cells were incubated with, Alexa Fluor 488 anti-rabbit donkey and Alexa Fluor 594 donkey secondary antibodies (Thermo Fisher Scientific) for 45 min in darkness. Then, HaCaT cells were incubated for 5 min in a DAPI solution (1:50.000) (Sigma-Aldrich) and then in 0.01% Tween PBS. Coverslips were immersed in a Dako mounting medium (Agilent).

Samples were mounted on glass slides. The images were acquired using Carl Zeiss LSM 700 (40X oil immersion optical lens) microscopes. The number of foci or the quantized immunofluorescent signals in the nuclei was measured with Fiji (ImageJ) software version 1.52 analyzed by GraphPad Prism 8.0.2 software (GraphPad, San Diego, CA, USA).

### 2.10. Western Blot Analysis

Western blot was carried out following Di martino et al. (2016) and Vivo et al. (2015) [35,36]. Immunoblots were revealed by means of specific antibodies. Antibodies against β-actin (C4) and NRF-2 (A-10) were from Santa Cruz Biotechnology, while p21WAF were from Invitrogen (Thermo Fisher Scientific). Each experiment was run in triplicate. The signal intensities of bands were quantified by Image Lab analysis software (Version Number 6.1, Biorad Laboratories, London, UK) and quantified by GraphPad Prism 8.0.2 software (GraphPad, San Diego, CA, USA).

## 3. Results

### 3.1. Evaluation of the Antioxidant Capacity of B. rapa Microgreens Exposed to Different Light Regimes

To analyze the effect of different light regimes (red–blue light and broad-spectrum white light) on the concentration of bioactive compounds in *Brassica rapa* L. microgreens, we evaluated the total polyphenols, flavonoids, and antioxidant capacity of the extracts from the microgreens grown under these two light conditions. The results of the FRAP assay, shown in Table 1, indicate that the total soluble antioxidant capacity of the microgreen extracts was significantly higher (*p* < 0.05) in samples exposed to RB light compared to those grown under W light. This shows that the red–blue light treatment enhanced the antioxidant capacity of the microgreens. Additionally, the concentration of total polyphenols and flavonoids was also found to be higher in microgreens grown under RB light in comparison to those grown under W light confirming that the RB light treatment was effective in increasing the levels of these phytochemicals in *Brassica rapa* L.

### 3.2. Cytotoxicity of B. rapa Microgreens MeOH Extract on Human Cells

**Figure 2:**
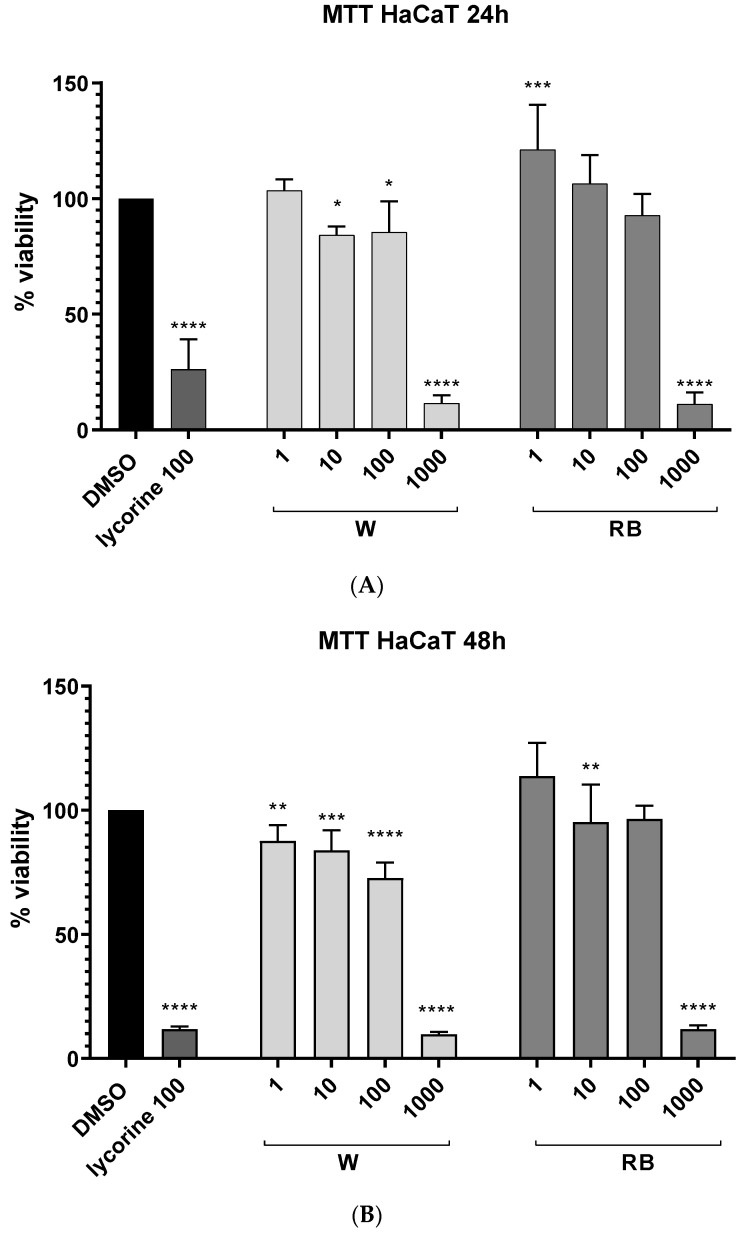
MTT viability test; on Y-axis the cell viability is showed in %. HaCaT cells were treated with the W (white light treatment) or RB (red–blue light treatment) organic extracts at the indicated concentrations for 24 h (**A**) and 48 h (**B**). Data are the means ± standard error (n = 6) of three independent biological replicates. Each mean was compared by one-way ANOVA, followed by Dunnett’s multiple comparisons test (*p*-value * < 0.01, ** < 0.05, *** *p* < 0.001; **** *p* < 0.0001). DMSO: cells in 0.1% DMSO, negative control; lycorine: positive control.

We analyzed the cytotoxic effect of W or RB methanol extracts obtained from *Brassica rapa* on human spontaneously immortalized HaCaT keratinocytes using the MTT assay. The number of viable cells was expressed as a percentage of negative control cells (DMSO 0.1%). A reduction in cell viability would show cytotoxic effects. The W or RB methanolic extracts were added to the cell culture medium at concentrations ranging from 1 to 1000 μg/mL. The cell cultures were incubated with the extracts for 24 and 48 h (Figure 2). After 24 h of incubation, the W extract at concentrations of 10 μg/mL and 100 μg/mL caused a reduction in HaCaT cell viability to 83% and 84%, respectively (Figure 2A). The cell viability was further reduced after 48 h of incubation (Figure 2B). Conversely, the RB extract at the lowest concentration used (1 μg/mL) caused a noticeable increase in cell viability (22–23%) at both 24 and 48 h of incubation (Figure 2). However, at the concentration of 1000 μg/mL, both extracts caused a dramatic reduction in cell viability like that detected by treating cells with 100 μM lycorine, used as a positive control of cytotoxicity. Based on the obtained results, we decided to perform further experiments using a concentration range from 1 to 100 μg/mL of both W and RB extract, and a 24 h incubation period.

### 3.3. Antioxidant Activity of B. rapa W and RB Extracts 

Next, we evaluated the antioxidant activities of *Brassica rapa* microgreens extracts in human HaCaT keratinocytes using the 2′-7′ dichlorofluorescein diacetate (DCFDA) assay. This assay measures ROS level in cells. Reactive oxygen species were induced in the cells using 1 mM H_2_O_2_ (3%).

Cells pretreated or not for 4 h with sub-toxic concentrations of RB and W extracts (from 1 to 100 μg/mL) were exposed to H_2_O_2_. TROLOX, a water-soluble permeable vitamin E analog, was used as a positive control for its known antioxidant activity. The negative control was treated with the vehicle alone (DMSO 0.1%) and was also used for diluting the extracts. 

**Figure 3:**
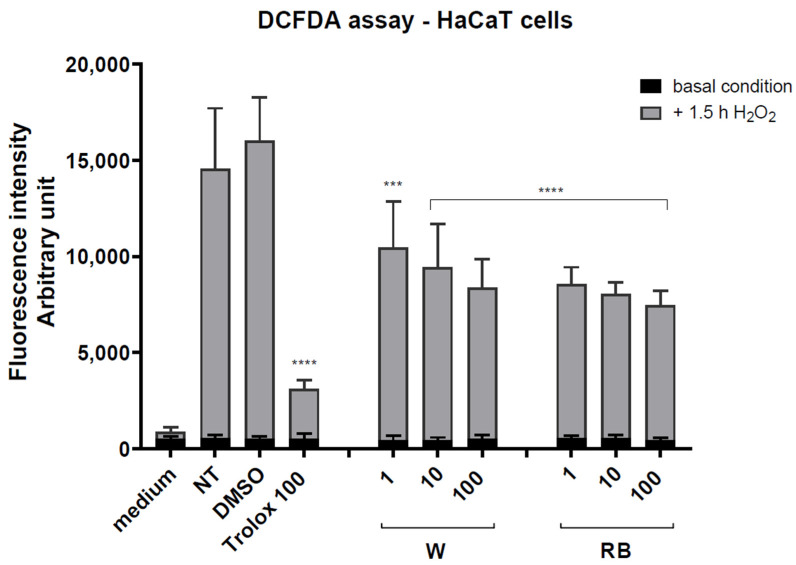
DCFDA antioxidant assay. HaCaT cells were seeded and pretreated with the indicated amount of W (white light treatment) or RB (red–blue light treatment) organic extract for 4 h. The DCFDA assay was performed on: medium (culture without cells used as background reference); NT (not-treated basal condition cells); DMSO (cells in 0.1% DMSO, negative control) and Trolox 100 μg/mL (positive control). All cell samples were treated with H_2_O_2_ (1 mM; 3%) for 1.5 h to induce ROS production. Data are the means ± standard error (n = 6) of three independent biological replicates. Statistical analysis was carried out by two-way ANOVA, followed by Tukey’s multiple comparison test (**** *p* < 0.001, *** *p* < 0.01).

The incubation of cells with increasing of the sole extract concentration did not significantly affect the basal level of intracellular ROS (Figure 3). However, pretreatment of cells with both W and RB extracts caused a notable reduction in ROS concentration induced by H_2_O_2_ (**** *p* < 0.001, *** *p* < 0.01). Interestingly, the RB extract proved higher efficiency in the quenching of ROS compared to the W extract, particularly at the lowest concentration applied (1 μg/mL) (Figure 3). These findings confirm that the microgreens grown under RB light conditions hold higher levels of antioxidant compounds that contribute to their enhanced antioxidant activity compared to microgreens grown under W light. However, at 10 μg/mL, the antioxidant effect reached a plateau and there was little added benefit in increasing the concentration of the extract up to 100 μg/mL) (Figure 3). 

### 3.4. Effect of W and RB Crude Extracts on Stress Granules Formation and DNA Damage

The effect of the extracts on oxidative stress-induced stress granule (SG) formation and DNA damage were tested in HaCaT cells by confocal immunofluorescence using antibodies against YB-1 (the Y-box binding protein 1) and γH2AX. 

We stimulated HaCaT cells, pretreated or not with W or RB microgreen extracts, with 300 μM H_2_O_2_ to induce oxidative stress and SGs assembly. As experimental controls, we used unstimulated cells and stimulated cells supplemented with the DMSO vehicle alone or with 100 μM Trolox as an antioxidant. 

**Figure 4:**
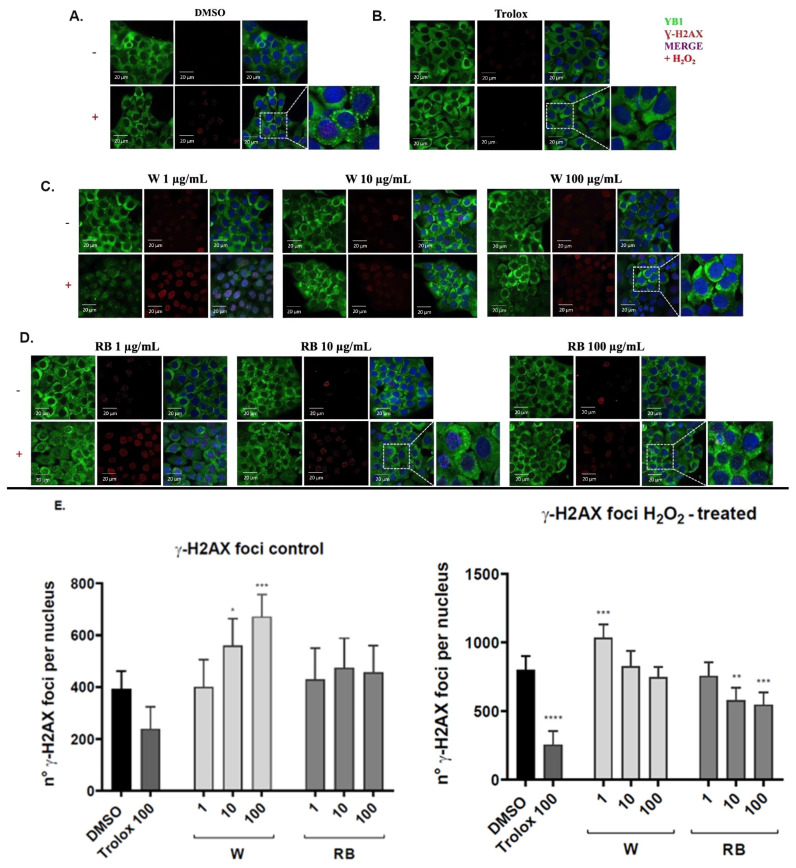
Confocal immunofluorescence to detect YB-1 subcellular localization and γ-H2AX foci in basal and oxidative stress conditions. (**A**–**D**) Confocal analysis of HaCaT cells treated with indicated concentrations of W (white light) and RB (red–blue light) extracts. Hydrogen peroxide was used to induce oxidative and/or genotoxic. Cells were stained with α-YB1 and α-γ-H2AX antibodies. Nuclei were stained with DAPI. (**E**) The quantitation of the foci number was performed by Fiji—ImageJ software version 1.52 and values were compared to the baseline/physiologic number of foci of the DMSO (cells in 0.1% DMSO, negative control) and Trolox 100 μg/mL (positive control). Data are the means ± standard error (n = 6) of three independent biological replicates. Statistical analysis was carried out by two-way ANOVA followed by Sidak’s multiple comparison tests (*p*-value * < 0.01, ** < 0.05, *** *p* < 0.001; **** *p* < 0.0001).

Hydrogen peroxide promoted the formation of YB-1 positive-SGs detectable as green spots in the cytoplasm and the assembly of γH2AX foci indicating the occurrence of DNA damage (Figure 4A,B,E). As expected, in cells treated with the antioxidant Trolox, SGs were undetectable, YB-1 was uniformly distributed into the cytoplasm (Figure 4B), and γH2AX foci were reduced compared to the control (Figure 4B,E).

In H_2_O_2_-unstimulated cells pretreated with the W or RB extract, SGs were undetectable. However, in cells treated with the W extract, the signal of γ-H2AX foci increased in a dose-dependent way, showing the occurrence of DNA damage. Conversely, in cells treated with the RB extract, the number of γ-H2AX foci was comparable to the control at all concentrations of RB extract used (Figure 4E, left panel). Upon oxidative stress, in W-treated cells, the number of γ-H2AX foci decreased in a dose-dependent way although it was higher compared to the control. Accordingly, YB-1 was completely nuclear at 1 μg/mL of W extract (Figure 4C,D) while it accumulated in the perinuclear region at 10 and 100 μg/mL of W extract. Conversely, in cells pretreated with 10 and 100 μg/mL of RB extract YB-1 was detected in SGs aggregates or distributed between the nucleus and cytoplasm while the number of γ-H2AX foci was significantly (* *p* < 0.01, ** *p* < 0.05, *** *p* < 0.001; **** *p* < 0.0001) reduced compared to the control thus indicating that the RB but not the W extract, was effective in protecting cells from oxidative stress-induced genotoxicity (Figure 4E). 

To deepen the protective role of the RB extract, we test both RB and W extracts on NRF2 (NF-E2-related factor 2) transcription factor and the cell cycle regulator p21WAF protein under unstressed conditions or in cells subjected to oxidative stress. W We analyzed the expression level of NRF2 and p21WAF protein by Western blot in control and H_2_O_2_-stimulated cells pretreated or not with W or RB extracts.

**Figure 5:**
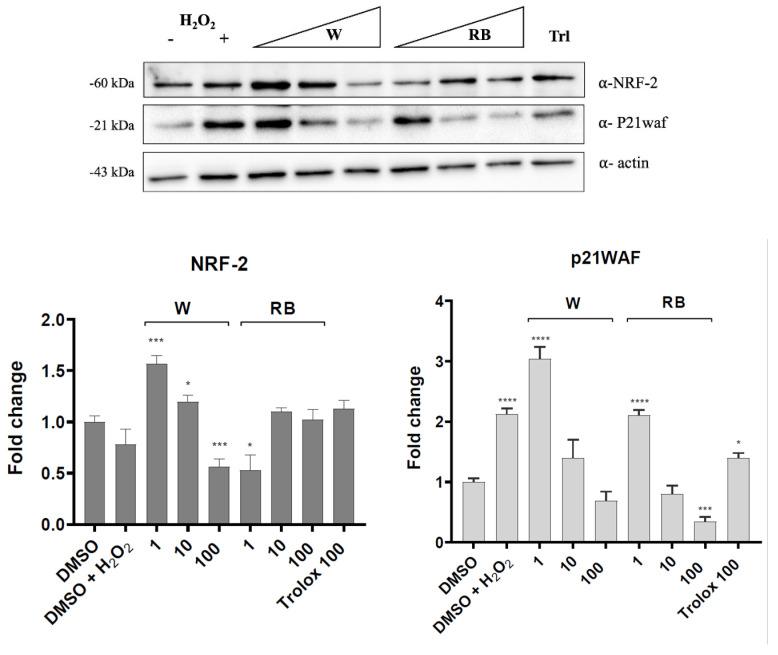

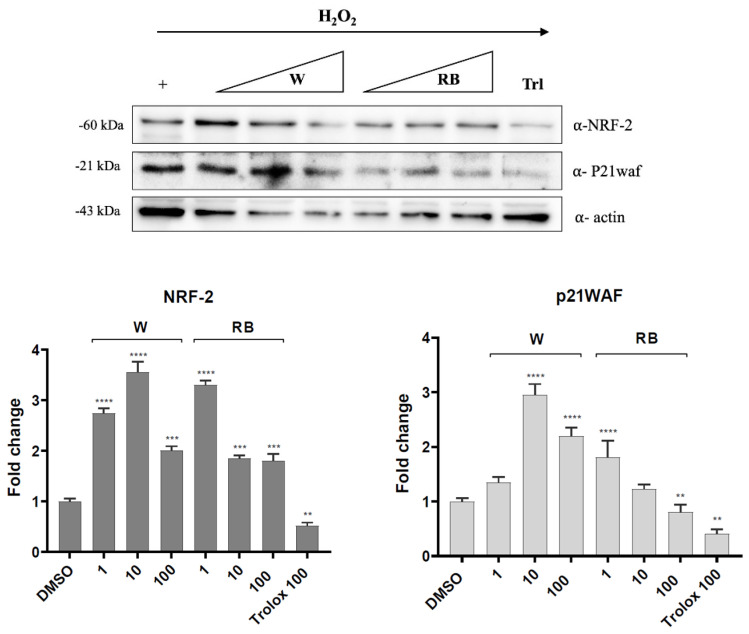
Western blot analysis of total extracts from HaCaT keratinocytes treated for 4 h with 1, 10 and 100 μg/mL of W (white light) and RB (red-blue light) extracts stimulated with H_2_O_2_ (**upper panel**) and without H_2_O_2_ (**lower panel**). DMSO (cells in 0.1% DMSO, negative control); DMSO + H_2_O_2_ (cells in 0.1% DMSO, negative control plus H_2_O_2_) and Trolox 100 μg/mL (positive control). Immunoblots were probed with NRF2 and p21WAF antibodies. β-actin was used as a loading control. The protein bands were quantified by ImageLab software version 4.1 (Bio-Rad, Hercules, CA, USA). Statistical analyses were carried out by two-way ANOVA followed by Sidak’s or Dunnett’s multiple comparison test (**** *p* < 0.0001, *** *p* < 0.001, ** *p* < 0.01, * *p* < 0.05).

Under unstressed conditions, pretreatment with low doses of W extract (1 and 10 μg/mL) upregulated NRF2 and p21WAF as a signal of activation of the cellular response to oxidative stress (Figure 5, upper panel). However, the highest concentration of W extract used (100 μg/mL) resulted in a dramatic decrease in NRF2 and p21WAF proteins (Figure 5, upper panel) thus suggesting that the toxic effect was prominent and negatively affected the pro-survival response of cells. This agreed with the data of the MTT assay indicating a reduced cell viability in this experimental condition (Figure 2). On the other hand, under basal conditions, RB extract did not affect NRF2 levels at all concentrations tested. The p21WAF protein was slightly induced only at the lowest concentration of RB, which shows a very mild activation of the oxidative stress response (Figure 5, upper panel). Following the oxidative stress, both W and RB extracts increased NRF2 protein levels (Figure 5, lower panel). 

## 4. Discussion

Microgreens are widely known as an excellent source of antioxidants and the choice of proper light treatment, during plant growth, especially enriched of red and blue wavelengths represents an efficient mean to regulate the synthesis of health-promoting molecules. Our study demonstrated that the *Brassica rapa* microgreens developed under W and RB light quality regimes exhibited a different modulation of phytochemicals.

Our data agree with previous studies on different crops. 

In blueberry fruit, the exposure to LED blue light (470 nm) significantly potentiates the total phenolic concentration [37,38]. Similar results have been obtained for brassica exposed at blue (660 nm) wavelength [39,40]. Other studies demonstrated that RB light boosted the production of polyphenols, anthocyanins, and flavonoids in strawberry [41] onion [21], and raspberry fruits [42].

Our results showed that RB light quality regimes strongly influence the concentration of phenolic compounds (total polyphenols and flavonoids) and the antioxidant capacity of *Brassica rapa*, confirming that the manipulation of the light spectrum is a promising approach for the modulation of antioxidant properties in vegetables. Light quality treatments is perceived by plants through specific photoreceptors [42]. Specifically, phytochrome (red light) and cryptochrome (blue light), are involved in the accumulation of phytochemicals [39,40]. We assume that red and blue light may play a significant role in the activation of specific biosynthetic pathways, crucial for the synthesis of phenolics such as phenylalanine ammonia-lyase (PAL), chalcone synthase flavanone-3-hydroxylase and anthocyanidin synthase [19]. Consistent with other studies [43,44] our results also showed that phenolic substances strongly contribute to the antioxidant activity of brassica seedlings. Indeed, total antioxidant capacity (FRAP) increased in RB compared to W microgreens.

Oxidative stress represents one of the key conditions triggering the formation of stress granules (SG), non-membrane-bound cytoplasmic aggregates of RNA and proteins [45,46]. The formation of stress granules (SGs) is a cellular strategy to face injuries caused by stress and enhance cell survival [45]. The oxidative stress can cause severe DNA damages whose extent determines an arrest of the cellular cycle and DNA repair or the activation of the apoptotic pathways [47]. YB-1 is a well-known oxidative stress sensor and a component of SGs. Hydrogen peroxide promoted the formation of YB-1 positive-SGs.

The YB-1 protein in cytoplasmic aggregates indicates the formation of SGs, while its accumulation in the nuclear compartment indicates the occurrence of DNA damage which is confirmed by histone γH2AX foci [48].

Inizio modulo

Our data suggest that, compared to RB, W extracts may have a higher concentration of cytotoxic pro-oxidant metabolites, which impair the correct assembly of stress granules. Accordingly to our hypothesis, in basal conditions in W-treated cells, SGs were not detected while the dose-dependent increase in γ-H2AX was evidence of the occurrence of DNA damage. Testing the antioxidant power of the brassica W and RB extract on human cells, we found intriguing results. More specifically, under basal conditions, conversely to the RB, the W extract seems to activate the nuclear factor NRF2, which represents a crucial regulator of cellular resistance to oxidative stress.

Both NRF2 and p21WAF proteins are known to be upregulated in response to oxidative stress, in order to protect cells from oxidative damage. In mammals, the NRF2 transcription factor modulates the expression of reactive oxygen species (ROS) detoxification enzymes to preserve cellular homeostasis [49], while the p21WAF protein promotes cell survival in response to oxidative stress [50].

Moreover, NRF-2 factor plays a pivotal role in maintaining the balance of redox homeostasis within cells by controlling a wide range of antioxidant enzymes and proteins involved in detoxification, such as superoxide dismutase (SOD), glutathione peroxidase (GPx), and catalase. Furthermore the NRF-2 also induces the expression of enzymes, such as glutathione-S-transferase (GST) and NAD(P)H quinone oxidoreductase 1 (NQO1). These enzymes work together to neutralize ROS and toxic electrophilic compounds, protecting cells from oxidative damage and maintaining cellular redox balance [51].

Our data indicate that, compared to control, W and RB extracts increased NRF2 protein levels under oxidative stress, suggesting that both extracts are active in triggering a cellular response against oxidative stress. However, the two extracts sorted different effects on the p21WAF cell cycle regulator. More specifically, only the RB extract was efficient in reducing p21WAF levels, indicating in RB-treated cells signs of recovery from oxidative stress facilitating cell cycle progression. On the other hand, the W extract did not significantly reduce p21WAF levels suggesting that the W extract was less efficient in helping cells recover from stress.

Our study demonstrated that the two extracts exert different effects on cells under oxidative stress: the RB extract promotes cell recovery and proliferation, while the W extract is not useful in cell protection against free radicals. Further studies would be needed to understand the underlying mechanisms of these differential effects on p21WAF regulation. 

Our data provide valuable insights into how extracts from plants grown in different light conditions may modulate cellular mechanisms related to stress granules and DNA damage. Understanding these aspects is crucial for assessing *B. rapa* microgreens’ effect on cellular health and may contribute to the identification of potential benefits in managing oxidative stress-related conditions. Our study also suggests that low concentrations of extracts are more effective than higher ones in free radical scavenging.

We can assume, as reported in [19], that compounds contributing to the antioxidant activity of RB extracts include some phenolics, namely flavones, flavanols, anthocyanidins, benzoic and cinnamic acids, which at specific concentrations—in our case, 1 μg/mL—may contrast and/or mitigate the DNA damage and the oxidative-induced genotoxicity. Identifying a method to boost such compounds in plant crude extract is worthy of attention because these molecules have powerful antioxidant, anti-inflammatory, and anti-cancer activity.

## 5. Conclusions

Our study demonstrated that the growth of *Brassica rapa* microgreens under light quality regimes white full spectra (W) and red, blue (RB) changes the concentrations of antioxidants in plant cells. In particular, the RB extract, according to its higher content of phenolic compounds and antioxidant activity, was more effective in reducing ROS levels and DNA damage compared to the W extract, particularly at the lowest applied concentration (1 μg/mL).

Our results also indicate that the effects obtained by RB extracts on human cells depend on the applied concentration, which has a critical role in determining beneficial or detrimental responses. This study may be relevant considering that controlling light spectrum is an eco-friendly, sustainable and safe approach to increase healthy compounds, because it does not require chemical or genetic manipulation. Plants richer in antioxidants may be used for the treatment of several diseases caused by oxidative stress with implications in the medical, nutraceutical, and biotechnological fields.

However, further investigation is required to fully understand the underlying mechanisms and to explore the broader applications of this approach. This could lead to further insights into the potential health benefits of microgreens and their optimal cultivation conditions.

## Figures and Tables

**Table 1:** Antioxidant capacity, total polyphenols, and total flavonoids. measured in microgreens of *Brassica rapa* L. grown for 15 days under W light (full spectrum) and RB light (60% R:40% B) regimes. Data are the means ± standard error (n = 8). Different letters indicate significant differences between conditions (*p* < 0.05).

	W Light Regime	RB Light Regime
Antioxidant capacity (µmol TE g^−1^ FW)	9.46 ± 0.11 ^b^	13.56 ± 0.16 ^a^
Total polyphenols (mg GAE g^−1^ FW)	2.96 ± 0.07 ^b^	4.45 ± 0.07 ^a^
Total flavonoids (mg CE g^−1^ FW)	3.86 ± 0.06 ^b^	4.40 ± 0.09 ^a^

TE: Trolox equivalent; GAE: Gallic Acid Equivalent; CE: Catechin Equivalent.

## Data Availability

Data are available from the corresponding author upon reasonable request.
